# *Protaetia brevitarsis* Extract Attenuates RANKL-Induced Osteoclastogenesis by Inhibiting the JNK/NF-κB/PLCγ2 Signaling Pathway

**DOI:** 10.3390/nu15143193

**Published:** 2023-07-19

**Authors:** Hye-Yeon Jang, Jeong-Mi Kim, Jong-Suk Kim, Byeong-Soo Kim, Young-Rae Lee, Jun Sang Bae

**Affiliations:** 1 Infectious Diseases Therapeutic Research Center, Korea Research Institute of Chemical Technology (KRICT), Daejeon 34114, Republic of Korea; janghyeyeon@naver.com; 2 Department of Biochemistry, Jeonbuk National University Medical School, 20 Geonji-ro, Deokjin, Jeonju 54907, Republic of Korea; wjdal4219@daum.net (J.-M.K.); jsukim@jbnu.ac.kr (J.-S.K.); 3 BK21FOUR 21st Century Medical Science Creative Human Resource Development Center, Jeonbuk National University, 567 Baekje-daero, Deokjin, Jeonju 54896, Republic of Korea; 4 Department of Companion and Laboratory Animal Science, Kongju National University, Yesan 32439, Republic of Korea; bskim@kongju.ac.kr; 5 Department of Oral Biochemistry, Institute of Biomaterials-Implant, School of Dentistry, Wonkwang University, 460, Iksan 54538, Republic of Korea; 6 Department of Pathology, College of Korean Medicine, Wonkwang University, 460, Iksan 54538, Republic of Korea

**Keywords:** *Protaetia brevitarsis*, edible insect, osteoclast differentiation, JNK, NF-κB, PLCγ2

## Abstract

*Protaetia brevitarsis* (PB)-derived bioactive substances have been used as food and medicine in many Asian countries because of their antioxidant, antidiabetic, anti-cancer, and hepatoprotective properties. However, the effect of PB extracts (PBE) on osteoclast differentiation is unclear. In this study, we investigated the effect of PBE on RANKL-induced osteoclastogenesis in mouse bone marrow-derived macrophages (BMMs). To investigate the cytotoxicity of PBE, the viability of BMMs was confirmed via MTT assay. Tartrate-resistant acid phosphatase (TRAP) staining and pit assays were performed to confirm the inhibitory effect of PBE on osteoclast differentiation and bone resorption. The expression levels of osteoclast differentiation-related genes and proteins were evaluated using quantitative real-time PCR and Western blotting. PBE attenuated osteoclastogenesis in BMMs in TRAP and pit assays without cytotoxicity. The expression levels of osteoclast marker genes and proteins induced by RANKL were decreased after PBE treatment. PBE suppressed osteoclastogenesis by inhibiting the RANKL-induced activated JNK/NF-κB/PLCγ2 signaling pathway and the expression of NFATc1 and c-Fos. Collectively, these results suggest that PBE could be a potential therapeutic strategy or functional product for osteoclast-related bone disease.

## 1. Introduction

Bone remodeling requires a balance between bone resorption by osteoclasts and bone formation by osteoblasts [1,2]. Excessive osteoclast differentiation and activation are associated with the development of bone diseases with low bone density and bone destruction, such as postmenopausal osteoporosis and rheumatoid arthritis, due to an imbalance between these cells [3,4,5]. Therefore, strategies to develop optimal treatments for these diseases should focus on the activation of osteoblasts or inhibition of osteoclasts.

Osteoclasts, known as cells with bone resorption function, are bone-specific multinucleated cells generated by proliferation, differentiation, and fusion of a monocyte/macrophage precursor lineage [1,6]. Osteoclast differentiation is controlled by various factors, including growth factors, cytokines, and hormones. An imbalance in these factors disrupts bone homeostasis and leads to bone pathogenesis [5,6,7,8].

Macrophage colony-stimulating factor (M-CSF) and receptor activator of nuclear factor-κB ligand (RANKL), produced by osteoblasts, play important roles in osteoclast differentiation. M-CSF and RANKL promote the proliferation and differentiation of osteoclast precursor cells, respectively [9,10]. RANKL activates TNF receptor-associated factor 6 (TRAF6) by binding to RANK expressed in osteoclasts, which further activates downstream signaling pathways, including phosphoinositide 3-kinase/serine-threonine protein kinase (PI3K/Akt), mitogen-activated protein kinases (MAPKs), and nuclear factor-κB (NF-κB). Eventually, it induces the activation of nuclear factors of activated T cells c1 (NFATc1) and c-Fos, which are key transcription factors involved in osteoclastogenic gene expression [11,12,13].

Insects have been regarded as functional foods and pharmaceutical resources [14]. Compounds derived from *Bruchidius dorsalis* have antioxidant properties [15]. Bioactive compounds, such as sesquiterpenoids and lactams, found in the edible insect *Aspongopus chinensis* Dallas, were effective against pain, dyspepsia, and kidney diseases [16]. *Scolopendra subspinipes mutilans*-derived small-molecule alkaloids exhibit anticoagulant activity [17]. Additionally, insects are used in traditional medicines to treat various conditions, including anemia, inflammation, hypertension, and asthma [18]. *Protaetia brevitarsis* (PB), belonging to the Cetoniidae family, is a white-spotted flower beetle that is widely distributed in East Asia (China, Japan, Korea, and Taiwan) and Europe [19]. Substances extracted from PB exhibit various bioactivities and are used for medicinal purposes in many Asian countries. In our previous study, PB protected islets from cytotoxicity in alloxan-treated pancreatic islets and db/db mice and exhibited antidiabetic efficacy by lowering plasma glucose levels and improving glucose tolerance, blood lipid parameters, and islet structure [20]. Fatty acids (palmitic acid, (Z)-9-octadecenoic acid and octadecenoic acid) extracted from the larvae of PB suggested anticancer effects by inducing apoptosis through potent cytotoxicity, DNA fragmentation, and caspase-3 activation against colon carcinoma cells [21]. A previous study reported that extracts from PB larvae fed a fermented aloe vera mixed diet against CCl_4_-induced hepatic injury in Sprague-Dawley rats showed potent hepatoprotective effects by increasing glutathione levels and inhibiting lipid peroxidation [22]. According to a recent report, oral administration of the PB larval extract to radiation-induced testicular injury male mice demonstrated testicular protective effects through reduction of germ cell apoptosis, improvement of sperm cell morphology and motility, and preservation of sperm count. In addition, it suppressed reactive oxygen species generation and exhibited antioxidative effects [23]. A recent study reported that PB extracts regulated bone remodeling in mice with OVX-induced osteoporosis and decreased RANKL-stimulated osteoclastogenesis by inhibiting p38 MAPK activation and NF-κB phosphorylation [24]. Also, PB-derived proteins showed antioxidant properties and promoted osteogenic differentiation in human bone marrow-derived mesenchymal stem cells [25]. Although PB exhibits various biological activities, its effect on bone remodeling, especially osteoclast differentiation, is not clearly known. Therefore, this study investigated the anti-osteoclastogenic effects of PB, which may provide a potential therapeutic strategy for bone diseases.

## 2. Materials and Methods

### 2.1. Samples and Reagents

*Protaetia brevitarsis* (PB) larvae were provided by Congmaeul. Ltd. (Imsil, Jeonbuk, Republic of Korea). The preparation and compositional analysis of PB extracts (PBE) were performed as previously described [20]. Briefly, the larvae were collected, washed with distilled water, and then lyophilized at −20 °C. The sample was then ground using an experimental pulverizer, and a powder was produced through a 30-mesh sieve. The powder of PB larvae was stored in an airtight container at −70 °C until the experiment. In addition, a gas chromatograph (GC 7890 B, Agilent Technologies, Inc., Santa Clara, CA, USA) equipped with a flame ionization detector was used for composition analysis of the PBE. Recombinant murine soluble RANKL and M-CSF were purchased from PeproTech (Rocky Hill, NJ, USA). JNK inhibitor SP600125 was purchased from Sigma-Aldrich (St. Louis, MO, USA).

### 2.2. Animals

Four specific pathogen-free 6- to 8-week-old male C57BL/6J mice (21–22 g) were purchased from Samtako Bio Korea, Inc. (Osan, Gyeonggi-do, Republic of Korea). Four animals were housed in a cage with 12-h light/dark cycles at 22 ± 2 °C and 50–60% humidity for 3 weeks. During the experimental period, general solid food (Purina Lab Rodent Chow #38057, Purina Co., Seoul, Republic of Korea) and filtered drinking water were supplied and changed every day for free intake. The euthanasia was performed under inhalation anesthesia (isoflurane, USP). All experiments were performed with bone marrow-derived macrophages (BMMs) isolated from C57BL/6J mice. All animal experiments were approved by the Animal Care and Use Committee of Wonkwang University (Approval No. WKU21-55).

### 2.3. Isolation and Differentiation of BMMs

Isolation and differentiation of BMMs were performed as described in a previous study [26]. Briefly, the cells were harvested by washing the bone marrow spaces of the mouse tibia and femur with phosphate-buffered saline (PBS), seeded in culture dishes, and cultured for 1 day. Thereafter, non-adherent cells were collected and incubated for 3 days in 𝛼-minimal essential medium (Gibco, Rockford, IL, USA) with 10% fetal bovine serum (Gibco) containing M-CSF (30 ng/mL). Then, the adherent cells were used as osteoclast precursors. To generate osteoclasts, BMMs were incubated with M-CSF (30 ng/mL) and RANKL (100 ng/mL) at 37 °C for 4 days.

### 2.4. Cell Viability Assay

Cell viability was determined using the 3-(4,5-dimethylthiazol-2-yl)-2,5-diphenyl tetrazolium bromide (MTT) (Sigma-Aldrich) assay. BMMs (1 × 10^4^ cells/well) were either seeded in 96-well culture plates with varying concentrations of PBE (0, 10, 25, 50, and 100 µg/mL) at 37 °C for 1 day or were treated with 50 µg/mL PBE under M-CSF treatment (30 ng/mL) for 4 days at 37 °C. After treatment, MTT solution (0.5 mg/mL in PBS) was added to each well for 30 min at 37 °C. Following incubation, formazan crystals were dissolved in DMSO (100 μL/well), and the optical density at 570 nm was measured using a microplate reader (Bio-Rad, Richmond, CA, USA). The MTT assays are performed at in least three independent experiments (*n* = 3).

### 2.5. Tartrate-Resistant Acid Phosphatase (TRAP) Staining and Activity

BMMs were treated with RANKL (100 ng/mL) and M-CSF (30 ng/mL) in the presence of various concentrations of PBE (0, 10, 25, 50, and 100 μg/mL) for 4 days. They were then fixed in 3.8% paraformaldehyde and stained for 1 h using a TRAP Kit (Sigma-Aldrich). The surface area of TRAP-positive polynucleated (nuclei > 3) cells was measured using the ImageJ software V 1.8.0 (NIH, Bethesda, MD, USA). To determine total TRAP activity, the collected cells were washed, lysed, and centrifuged. *p*-Nitrophenylphosphate (Sigma-Aldrich) was used as a substrate for TRAP, and optical density was measured at 405 nm using a microplate reader (Bio-Rad). The experiments were performed in at least three independent experiments (*n* = 3).

### 2.6. Pit Assay

BMMs were seeded in an osteo assay 48-well plate (COSMO BIO, Carlsbad, CA, USA) at a density of 3 × 10^4^ cells per well, and the cells were treated with RANKL (100 ng/mL) and M-CSF (30 ng/mL) in the presence of PBE for 4 days. The cells were completely removed, washed with distilled water, and dried at room temperature. The bone resorption area was observed using a light microscope (magnification, ×50) and calculated using the ImageJ software V 1.8.0 (NIH). The experiments were performed in at least three independent experiments (*n* = 3).

### 2.7. RNA Extraction and Quantitative Real-Time PCR (qRT-PCR) Assay

BMMs treated with or without PBE (50 µg/mL) were cultured in the presence of M-CSF (30 ng/mL) and RANKL (100 ng/mL) for 4 days. The cultured cells were washed with cold PBS, and total RNA was extracted using TRIzol reagent (Invitrogen, Carlsbad, CA, USA) on the indicated days. Reverse transcription of 1 μg of total RNA was performed using a PrimeScript™ RT reagent kit (TaKaRa Bio, Shiga, Japan) according to the manufacturer’s instructions. The ABI Prism 7900 Sequence Detection System (Applied Biosystems, Foster City, CA, USA) and SYBR Green polymerase chain reaction Master Mix (Applied Biosystems) were used for qRT-PCR. For CT value analysis, the relative gene expression level was calculated using the 2^−ΔΔCt^ method [27]. The results were normalized to the expression of the GAPDH reference housekeeping gene. The experiments were performed in at least three independent experiments (*n* = 3). The primer sequences used for qRT-PCR are listed in Table 1.

### 2.8. Western Blot Analysis

Briefly, lysates were obtained by lysing BMMs in RIPA lysis buffer (Pierce Biotechnology, Rockford, IL, USA). Protein concentrations were determined using the Bradford assay (Bio-Rad). Each protein sample was separated using sodium dodecyl sulfate-polyacrylamide gel electrophoresis and transferred onto polyvinylidene difluoride membranes (GE, Buckinghamshire, UK). Each membrane was blocked with 5% skimmed milk or 5% bovine serum albumin for 1 h, probed with primary antibodies, and incubated overnight at 4 °C. The membranes were washed with tris-buffered saline containing Tween 20 and treated with secondary antibodies containing horseradish peroxidase for 1 h. Protein levels were detected using a LAS-4000 image analyzer (FujiFilm, Tokyo, Japan). Primary antibodies against extracellular signal-regulated kinase (ERK), *p*-ERK, p38, *p*-p38, c-Jun-*N*-terminal kinase (JNK), *p*-JNK, p65, *p*-p65, IκBα, *p*-IκBα, *p*-PLCγ2, CREB, *p*-CREB, and c-Fos were purchased from Cell Signaling Technology (Danvers, MA, USA). Anti-PLCγ2 and anti-NFATc1 antibodies were purchased from Santa Cruz Biotechnology (Dallas, TX, USA). The anti-β-actin antibody was purchased from Sigma-Aldrich. 

### 2.9. Statistical Analysis

Data are expressed as the mean ± standard deviation of results from at least three independent experiments. Statistical analyses were performed using ANOVA and Duncan’s test. Statistical significance was set at *p* < 0.05.

## 3. Results

### 3.1. Effects of PBE on BMM Viability

**Figure 1:**
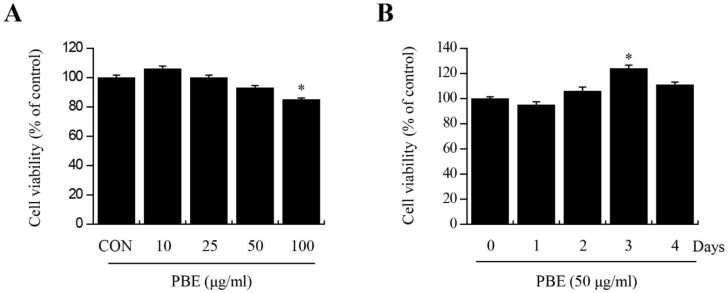
Effects of PBE on BMM viability. (**A**) BMMs (1 × 10^4^ cells/well) were seeded in 96-well plates, treated with PBE (0, 10, 25, 50, and 100 µg/mL), and incubated at 37 °C with 5% CO_2_ for 24 h. (**B**) BMMs were treated with PBE (50 µg/mL) and incubated for 0, 1, 2, 3, or 4 days. After incubation, cell viability was measured using the MTT assay. Bars labeled with different superscripts indicate significant differences (* *p* < 0.05 vs. control or vs. 0 days). The results are expressed as the mean ± standard deviation of at least three independent experiments (*n* = 3).

To confirm the cytotoxicity of PBE against BMMs, cells were incubated with various concentrations of PBE (0, 10, 25, 50, and 100 μg/mL) for 24 h and assessed using the MTT assay. When the PBE concentration was 50 μg/mL or lower, there was no effect on the viability of BMMs (Figure 1A). In addition, cell viability was confirmed to be time-dependent at a PBE concentration of 50 μg/mL, and there was no significant difference in the viability of BMMs treated with PBE (Figure 1B). Based on these results, 50 μg/mL of PBE was used for further experiments.

### 3.2. PBE Suppressed RANKL-Induced Osteoclast Differentiation and Bone Resorption

**Figure 2:**
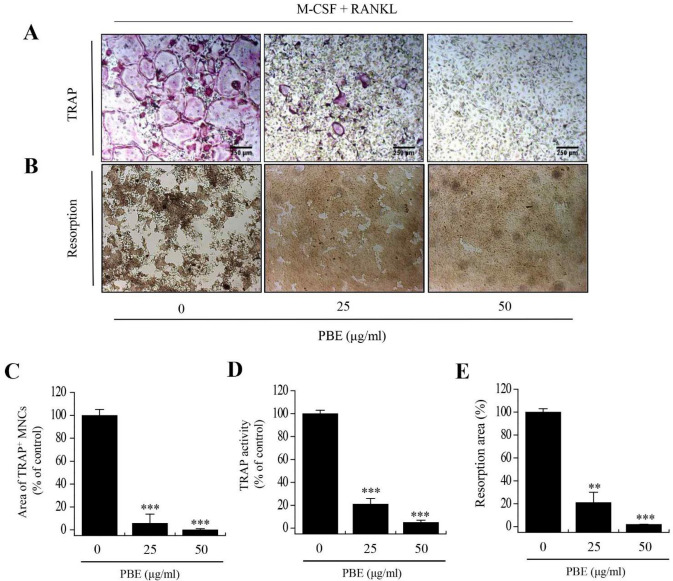
Effects of PBE on RANKL-induced osteoclast differentiation and bone resorption. BMMs were cultured for 4 days in the presence of RANKL (100 ng/mL) and M-CSF (30 ng/mL) with PBE (0, 25, and 50 μg/mL). (**A**) TRAP staining (upper panel) and (**B**) bone resorption (lower panel) were performed to visualize osteoclast differentiation. Scale bar, 250 μm. (**C**) TRAP-positive MNCs (TRAP^+^ MNCs; nuclei > 3) were counted as mature osteoclasts. (**D**) TRAP activity in BMMs was analyzed on day 4 according to the manufacturer’s protocol. (**E**) Bone resorption areas were quantified using ImageJ software, and significant differences between the PBE and control groups are indicated (** *p* < 0.005; *** *p* < 0.001). Data are presented as the mean ± standard deviation and are representative of at least three experiments.

To evaluate the inhibitory effects of PBE on osteoclastogenesis, the cells were incubated with RANKL and PBE (0, 25, and 50 μg/mL) for 4 days. To confirm osteoclast differentiation, TRAP staining (Figure 2A), TRAP^+^ multinucleated cell (MNC) counting (Figure 2C) and TRAP activity assays (Figure 2D) were performed. In the RANKL-treated group, the formation of TRAP-positive MNCs was observed, in which cells aggregated, bundled, and increased the activation of TRAP, thereby showing that BMMs were fully differentiated into osteoclasts. However, multinucleated osteoclast differentiation and TRAP activity were significantly reduced by PBE in a dose-dependent manner. Osteoclasts have the ability to resorb mineralized matrix and form resorption pits. Therefore, we investigated whether the inhibition of osteoclast formation by PBE affects bone resorption. As shown in Figure 2B,E, RANKL induced bone resorption pits in osteoclasts, and PBE decreased the area of the bone resorption pits in a concentration-dependent manner. These results suggest that PBE inhibited osteoclastic differentiation and bone resorption.

### 3.3. PBE Down-Regulated the Expression of Osteoclastogenesis-Related Genes

**Figure 3:**
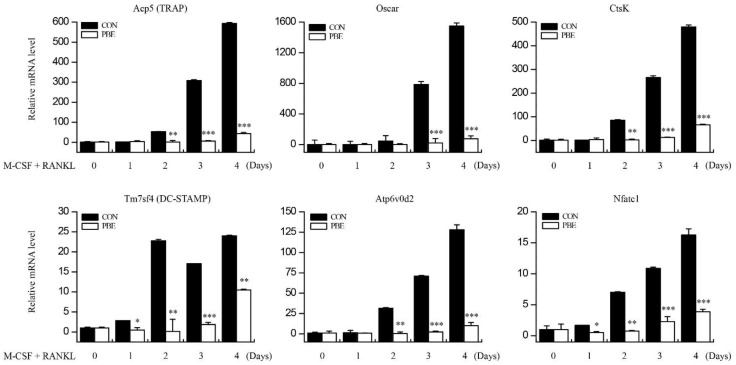
Effect of PBE on the expression of osteoclastogenesis-related genes. BMMs were cultured with RANKL (100 ng/mL) and M-CSF (30 ng/mL) in the absence or presence of PBE (50 μg/mL) for 0, 1, 2, 3, or 4 days. The expression of genes (Acp5 (TRAP), Oscar, CTSK (cathepsin K), Tm7sf4 (dendritic cell-specific transmembrane protein, DC-STAMP), Atp6v0d2, and NFATc1) involved in osteoclast differentiation was evaluated by qRT-PCR. Experiments were performed in triplicate, and the error bars represent the standard deviation. Significant differences between the PBE and control groups are indicated (* *p* < 0.05; ** *p* < 0.005; *** *p* < 0.001).

Based on the results of the inhibition of osteoclast differentiation by PBE, the expression of mRNA involved in osteoclast differentiation was evaluated by qRT-PCR. RANKL stimulation increased the mRNA expression of Acp5 (TRAP), Oscar, CTSK (cathepsin K), Tm7sf4 (dendritic cell-specific transmembrane protein, DC-STAMP), Atp6v0d2, and Nfatc1 in BMMs. However, PBE treatment considerably decreased the mRNA expression of these genes (Figure 3). These results suggest that PBE may affect the signaling pathways in the early stages of osteoclastogenesis.

### 3.4. PBE Inhibits MAPK and NF-κB Activation in BMMs

**Figure 4:**
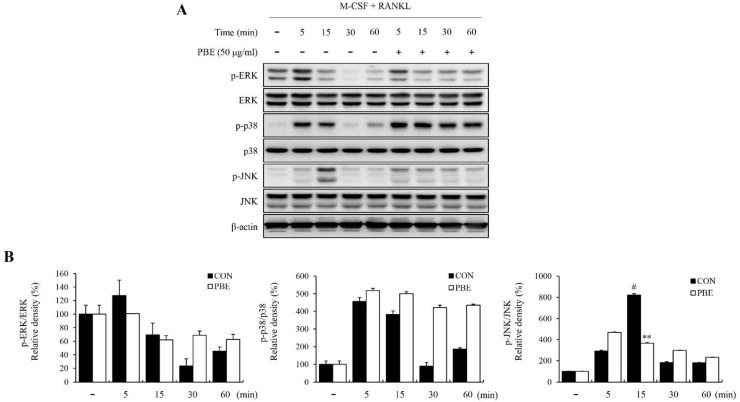
Effect of PBE on MAPK activation in BMMs. BMMs were cultured with RANKL (100 ng/mL) and M-CSF (30 ng/mL), with or without PBE (50 μg/mL), for 5, 15, 30, and 60 min. (**A**) Protein expression levels were analyzed by Western blotting using anti-ERK, *p*-ERK, p38, *p*-p38, JNK, and *p*-JNK antibodies. (**B**) Band intensities were quantified using ImageJ software. Bars labeled with different superscripts indicate significant differences (# *p* < 0.05 vs. RANKL-non treated group; ** *p* < 0.005 vs. control). Data are expressed as the mean ± SD of at least three independent experiments (*n* = 3).

**Figure 5:**
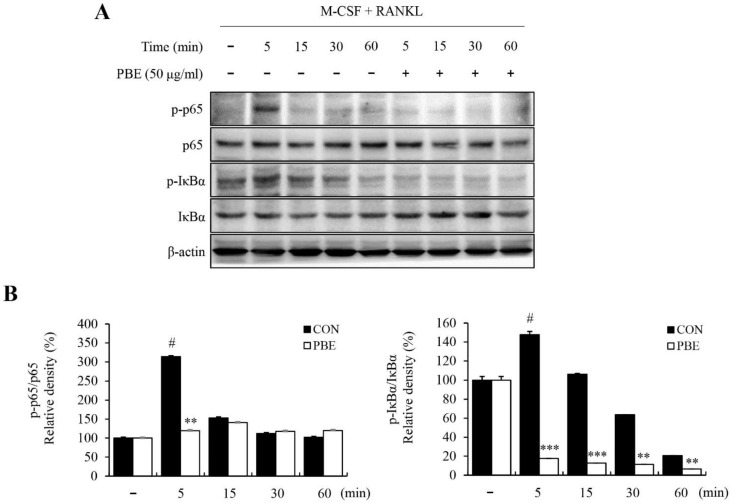
Effect of PBE on NF-κB activation in BMMs. BMMs were cultured with RANKL (100 ng/mL) and M-CSF (30 ng/mL), with or without PBE (50 μg/mL), for 5, 15, 30, and 60 min. (**A**) Protein expression levels were analyzed by Western blotting using anti-NF-κB p65, *p*-NF-κB p65, IκBα, and *p*-IκBα antibodies. (**B**) Image J software was used to quantify the protein bands. Bars labeled with different superscripts indicate significant differences (# *p* < 0.05 vs. RANKL non-treated group; ** *p* < 0.005 vs. control; *** *p* < 0.001 vs. control). Data are presented as the mean ± SD of at least three independent experiments (*n* = 3).

M-CSF and RANKL induce the MAPK and NF-κB signaling pathways as early signaling pathways during osteoclast proliferation and differentiation, as well as bone resorption. Therefore, we examined whether PBE could inhibit the activation of MAPK (ERK, p38, and JNK) and NF-κB in BMMs by Western blot analysis. As shown in Figure 4A, PBE significantly suppressed the phosphorylation of JNK, which was increased by M-CSF and RANKL, but had no significant effect on ERK and p38. In addition, PBE inhibited RANKL-stimulated NF-κB, p65, and IκBα phosphorylation (Figure 5A). The expression of each protein was normalized, and the relative expression levels were analyzed (Figure 4B and Figure 5B). These results show that PBE inhibited osteoclast differentiation by downregulating the activation of JNK and NF-κB signaling pathways.

### 3.5. PBE Regulates NFATc1 and c-Fos Expression by Inhibiting the JNK/NF-κB/PLCγ2 Signaling Pathway

**Figure 6:**
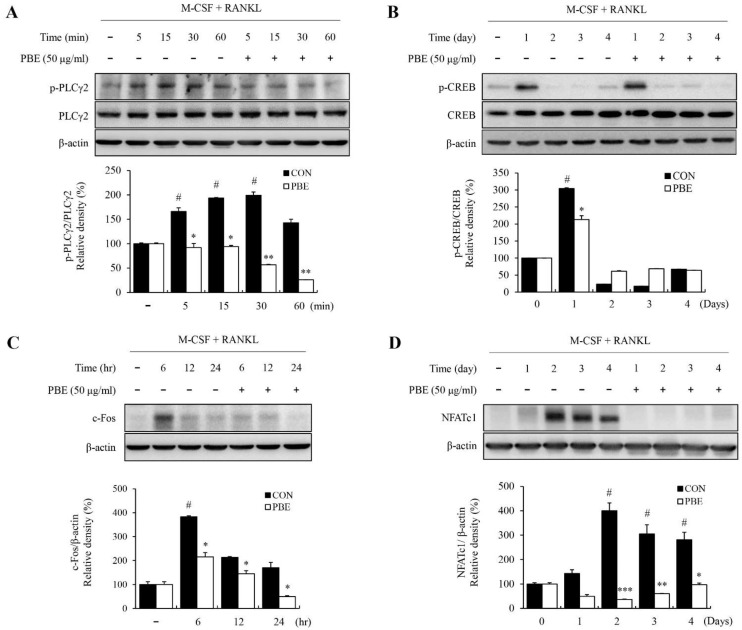
Effect of PBE on RANKL-induced PLCγ2, c-Fos, and NFATc1 expression. BMMs were cultured with RANKL (100 ng/mL) and M-CSF (30 ng/mL), with or without PBE (50 μg/mL), for the indicated time periods. Western blotting was performed to analyze the protein expression levels of (**A**) PLCγ2, *p*-PLCγ2, (**B**) CREB, *p*-CREB, (**C**) c-Fos, and (**D**) NFATc1. Protein expression levels were quantified using ImageJ software, and values are expressed as the mean ± SD (*n* = 3). Bars labeled with different superscripts indicate # *p* < 0.05 vs. RANKL-non treated group; * *p* < 0.01 vs. control; ** *p* < 0.005 vs. control; *** *p* < 0.001 vs. control.

**Figure 7:**
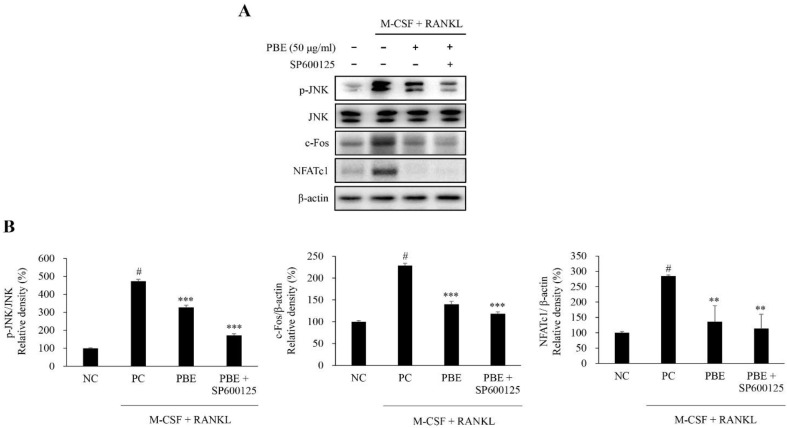
Inhibitory effect of PBE on osteoclastogenesis is mediated through the JNK signaling pathway. BMMs were cultured with RANKL (100 ng/mL) and M-CSF (30 ng/mL) for 4 days. Then, the cells were pretreated with the JNK inhibitor SP600125 (20 μM) for 30 min, followed by stimulation with or without PBE at a concentration of 50 μM. (**A**) Western blotting was performed to analyze the protein expression levels of *p*-JNK, JNK, c-Fos, and NFATc1. (**B**) Protein bands were quantified using Image J software. Bars labeled with different subscripts indicate significant differences (# *p* < 0.001 vs. RANKL untreated group (NC; normal control), ** *p* < 0.005 vs. RANKL treated group (PC; positive control), *** *p* < 0.001 vs. PC). Data are presented as mean ± SD from at least three independent experiments (*n* = 3).

M-CSF and RANKL-induced MAPK, NF-κB, phospholipase C gamma 2 (PLCγ2), and CREB activation are known to activate the osteoclast differentiation factors c-Fos and NFATc1. Therefore, we investigated whether PBE could affect the expression of PLCγ2 and CREB, which are important signaling pathways for RANKL-induced osteoclast differentiation. Our results confirm that PBE significantly reduced the phosphorylation levels of PLCγ2 and CREB during RANKL-induced osteoclast differentiation (Figure 6A,B). Next, we investigated whether PBE affected the protein expression levels of c-Fos and NFATc1, which are key transcription factors for RANKL-induced osteoclast differentiation. Stimulation with M-CSF and RANKL increased the protein expression levels of c-Fos and NFATc1, and these protein levels, which increased upon treatment with PBE, were significantly reduced (Figure 6C,D). Additionally, to confirm that the inhibitory effect of PBE on osteoclastogenesis is mediated through the JNK signaling pathway, we investigated the phosphorylation level of JNK by co-treating PBE with the JNK inhibitor SP600125. As a result, it was confirmed that the phosphorylation level of JNK increased by M-CSF and RANKL was decreased when treated with PBE, and the phosphorylated level of JNK was lowered more when co-treated with PBE and SP600125. To investigate whether c-Fos and NFATc1, known as the final steps in the signaling pathway of osteoclastogenesis, are regulated by the JNK signaling pathway, we simultaneously treated PBE and SP600125, and examined the protein expression levels of c-Fos and NFATc1. The protein expression levels of c-Fos and NFATc1 were increased by M-CSF and RANKL, but were reduced upon treatment with PBE. Furthermore, the protein expression levels of c-Fos and NFATc1 were more decreased when co-treated with PBE and SP600125 (Figure 7A). The expression of each protein was normalized, and the relative expression levels were analyzed (Figure 7B). Taken together, these results suggest that PBE mediated the inhibition of NFATc1 and c-Fos expression via various signaling pathways, such as the JNK/NF-κB/PLCγ2 signaling pathway, during osteoclast differentiation.

## 4. Discussion

Bone remodeling occurs as a balance between osteoblast and osteoclast differentiation [1,2]. However, when an imbalance occurs, dysregulation of bone homeostasis occurs, which can cause various pathological conditions, such as osteoporosis and rheumatoid arthritis [3,4,5], primarily resulting from the overexpression of osteoclast activity. Therefore, inhibition of osteoclast activity may be a major therapeutic strategy for these diseases. Recently, various drugs, such as bisphosphonates and estrogens, have been used as anti-osteoporosis drugs, but they have some side effects, such as vaginal bleeding, deep vein embolism, breast cancer, atypical femoral fractures, and necrosis of the jaw [28,29,30,31]. Currently, as an alternative treatment strategy to compensate for these side effects, the focus is on discovering insects that are regarded as functional food and pharmaceutical resources.

Many insect-derived bioactive substances are known to be used as antioxidants, anticoagulants, indigestion, inflammation, and hypertension [15,16,17,18]. Among them, bioactive substances derived from PB, an edible insect, have been used as a medicinal and functional food in many Asian countries due to their antioxidant, antidiabetic, anticancer, hepatoprotective, and testicular protective effects [20,21,22,23]. As a result of the component analysis of PB larvae in previous studies, oleic acid and volatile compounds were identified, and oleic acid was the most predominant fatty acid [19,20]. The PB used in this study contained a large amount of oleic acid, and it contained 3.402 μg of oleic acid per 50 μg of PB powder (Figure S1). Oleic acid is known to perform various bioactivities [32,33]. In a recent study, oleic acid was reported to be a potential inducer of bone formation by enhancing the differentiation and proliferation of human adipose tissue cells, increasing calcium deposits, and promoting bone formation by improving cell interactions [34]. Previous studies have reported that dietary supplementation, including dairy drinks rich in oleic acid, fatty acids, calcium, and vitamins, improves some bone metabolism biomarkers in postmenopausal healthy women [35]. Therefore, in this study, we investigated the effect of PBE on osteoclastogenesis.

In this study, we show that PBE can effectively reduce the number of osteoclasts and the bone resorption area, which was confirmed by the downregulation of the expression of marker genes related to osteoclast differentiation. Additionally, PBE suppressed RANKL-induced NFATc1 and c-Fos expression by inhibiting the phosphorylation of JNK and NF-κB. Collectively, these results suggest that PBE attenuates osteoclastogenesis via the JNK/NF-κB signaling pathway.

M-CSF and RANKL are important cytokines for osteoclast differentiation and are known to induce osteoclast formation in vitro [36,37,38]. Consistent with previous studies, this study shows that RANKL stimulation induced the formation of osteoclasts and bone resorption pits. However, it reduced RANKL-induced osteoclast formation, as the number of TRAP-positive MNCs, TRAP activity, and bone resorption in BMMs decreased after PBE treatment. These results suggest that PBE inhibits osteoclast differentiation and bone resorption.

In addition, RANKL induces the development of mature osteoclasts by inducing the expression of multiple osteoclast-specific genes [1]. The expression of genes related to osteoclast differentiation, such as Acp5, Oscar, CTSK, Tm7sf4, Atp6v0d2, c-Fos, and NFATc1, is closely related to differentiation and function [1,39,40]. Consistent with these results, the mRNA expression levels of Acp5, Oscar, CTSK, Tm7sf4, Atp6v0d2, and NFATc1 were upregulated by RANKL in our study. However, the increased mRNA expression levels of these genes were reduced by PBE treatment. These results suggest that the inhibitory effect of PBE on osteoclast differentiation and bone resorption occurs through inhibition of the expression of activated osteoclast-specific genes.

The signal transduction pathways in osteoclastogenesis form diverse, complex networks. The MAPK and NF-κB families are known to be important signaling pathways for osteoclastogenesis. M-CSF and RANKL act through the activation of MAPK, consisting of ERK, p38, and JNK signaling, during osteoclast differentiation and bone resorption [1,41]. Moreover, recent studies have reported that the activation of MAPK by M-CSF or RANKL differs in terms of the extent, duration, and isotype specificity of MAPK phosphorylation [41,42]. In this study, we confirm that PBE or co-treatment with PBE and JNK inhibitors decreases the phosphorylation level of JNK, which was increased by RANKL, but does not affect the phosphorylation of ERK and p38. Previous studies have reported that RANKL stimulates JNK to induce activation of the transcription factors c-Jun, c-Fos, and NFATc1, and blocking the JNK signaling pathway inhibits RANKL-induced osteoclast differentiation [41,42,43,44]. These results suggest that PBE may inhibit osteoclast differentiation by regulating the phosphorylation of JNK.

The NF-κB signaling pathway, which is activated by the interaction between RANKL and RANK receptors, plays an important role in osteoclastogenesis [11,45]. NF-κB signaling is activated by various stimuli, including RANKL, TNF-α, bacterial endotoxins, toll-like receptor ligands, CD40L, and interleukin-1. These stimuli usually induce complexes via TRAF and activate the IκB kinase complex. The activated kinase complex phosphorylates the NF-κB inhibitory protein IκBα, which undergoes proteasomal degradation, resulting in nuclear translocation and activation of various NF-κB dimers [11,45]. Previous studies have demonstrated that RANKL induces osteoclast differentiation through the phosphorylation of NF-κB and proteasomal degradation of IκBα [46,47,48]. In addition, activation of NF-κB p65 by RANKL regulation promotes transcription of osteoclastogenesis-related genes by translocating p65 to the cell nucleus and binding to the NFATc1 promoter [49]. Consistent with previous studies, our results show that the phosphorylation levels of IκBα and NF-κB p65 were increased by RANKL. However, the phosphorylation levels of these proteins decreased after PBE treatment. These results suggest that PBE may decrease osteoclastogenesis by inhibiting the NF-κB pathway.

RANKL-induced MAPK and NF-κB activation activate the osteoclast differentiation factors c-Fos and NFATc1, which are important for osteoclast differentiation and activity. Also, c-Fos and NFATc1 are known to be the final steps in the MAPKs- and NF-κB-related osteoclastogenesis signaling pathways [41,45]. NFATc1, a master regulator of osteoclast differentiation, is activated by RANKL and plays an essential role in osteoclastogenesis [50,51,52,53]. NFATc1 is a well-known key transcription factor for c-Fos, which plays an essential role in osteoclastogenesis induced by M-CSF and RANKL [52,53]. In this study, we show that PBE or co-treatment with PBE and JNK inhibitors suppressed the expression of NFATc1 and c-Fos, which are key regulators of osteoclasts. Based on previous studies and the present study, we suggest that PBE can inhibit osteoclast differentiation by reducing the expression levels of NFATc1 and c-Fos induced by RANKL. Additionally, the PLCγ2-calcium ion (Ca^2+^) signaling pathway also plays an important role in osteoclast differentiation. Previous studies have reported that the RANKL–RANK interaction activates PLCγ2-Ca^2+^ signaling, which in turn activates CREB or NFATc1 [54,55,56,57]. Therefore, we investigated whether PBE affects PLCγ2 and CREB activation. In this study, PBE reduced the phosphorylation of PLC𝛾2 and CREB, which are important for osteoclast differentiation, followed by the repression of c-Fos and NFATc1 expression. In addition, the combined treatment of PBE and SP600125 was found to reduce the expression levels of c-Fos and NFATc1 proteins more than that of PBE alone. These results suggest that PBE regulates osteoclastogenesis by inhibiting RANKL-induced PLC𝛾2-CREB signaling pathway activation. However, further studies are needed to clarify the role of PBE in osteoclastogenesis and bone disease. In particular, it is necessary to confirm various clinical data related to bone formation by PBE through animal studies. In this study, in vivo studies were not conducted; however, in vitro experiments may help understand the role of PBE in the bone remodeling process. Taken together, although there are some limitations, this is the first study to demonstrate that PBE suppresses the JNK/NF-κB/PLCγ2 signaling pathway and reduces the expression levels of NFATc1 and c-Fos involved in osteoclastogenesis, thereby regulating RANKL-induced osteoclastogenesis.

## 5. Conclusions

In conclusion, our results demonstrate the inhibitory effect of PBE on osteoclast differentiation and bone resorption. PBE reduced the levels of *p*-JNK, *p*-NF-κB, and *p*-PLCγ2 proteins in RANKL-induced BMMs, thus reducing the expression levels of NFATc1 and c-Fos. Therefore, this study suggests that PBE inhibits osteoclastogenesis through the JNK/NF-κB/PLCγ2 signaling pathway and could be a potential therapeutic strategy or functional product for osteoclast-related bone diseases.

## Figures and Tables

**Table 1:** Primer sequences used for qRT-PCR.

Gene Names	Primer Sequences
Mouse *ACP5* (TRAP)	forward: 5′-CTGGAGTGCACGATGCCAGCGACA-3′ reverse: 5′-TCCGTGCTCGGCGATGGACCAGA-3′
*Oscar*	forward: 5′-GGGGTAACGGATCAGCTCCCCAGA-3′ reverse: 5′-CCAAGGAGCCAGAACGTCGAAACT-3′
*CTSK*	forward: 5′-ACGGAGGCATTGACTCTGAAGATG-3′ reverse: 5′-GTTGTTCTTATTCCGAGCCAAGAG-3′
*TM7SF4 (DC-STAMP)*	forward: 5′-TGGAAGTTCACTTGAAACTACGTG-3′ reverse: 5′-CTCGGTTTCCCGTCAGCCTCTCTC-3′
*ATP6V0D2*	forward: 5′-TCAGATCTCTTCAAGGCTGTGCTG-3′ reverse: 5′-GTGCCAAATGAGTTCAGAGTGATG-3′
*NFATc1*	forward: 5′-CTCGAAAGACAGCACTGGAGCAT-3′ reverse: 5′-CGGCTGCCTTCCGTCTCATAG-3′;
*GAPDH*	forward: 5′-TGCCAGCCTCGTCCCGTAGAC-3′ reverse: 5′-CCTCACCCCATTTGATGTTAG-3′

TRAP, tartrate-resistant acid phosphatase; *Oscar*, osteoclast-associated immunoglobulin-like receptor; *CTSK*, cathepsin K; *DC-STAMP*, dendritic cell-specific transmembrane protein; *ATP6V0D2*, ATPase H+ transporting V0 subunit D2; *NFATc1*, nuclear factor of activated T cells 1; *GAPDH*, glyceraldehyde-3-phosphate dehydrogenase.

## Data Availability

The data are available from the corresponding author upon reasonable request.

## Supplementary Materials

The following supporting information can be downloaded at: https://www.mdpi.com/article/10.3390/nu15143193/s1, Figure S1: Gas chromatography profile of Protaetia brevitarsis. Oleic acid was detected.

## References

[1] Boyle W.J., Simonet W.S., Lacey D.L. (2003). Osteoclast differentiation and activation. Nature.

[2] Chen X., Wang Z., Duan N., Zhu G., Schwarz E.M., Xie C. (2018). Osteoblast-osteoclast interactions. Connect. Tissue Res..

[3] Akiyama T., Dass C.R., Choong P.F. (2008). Novel therapeutic strategy for osteosarcoma targeting osteoclast differentiation, bone-resorbing activity, and apoptosis pathway. Mol. Cancer Ther..

[4] Teitelbaum S.L. (2000). Bone resorption by osteoclasts. Science.

[5] Rodan G.A., Martin T.J. (2000). Therapeutic approaches to bone diseases. Science.

[6] Ono T., Nakashima T. (2018). Recent advances in osteoclast biology. Histochem. Cell Biol..

[7] Manolagas S.C. (1995). Role of cytokines in bone resorption. Bone.

[8] Han Y., You X., Xing W., Zhang Z., Zou W. (2018). Paracrine and endocrine actions of bone-the functions of secretory proteins from osteoblasts, osteocytes, and osteoclasts. Bone Res..

[9] Hedvičáková V., Žižková R., Buzgo M., Rampichová M., Filová E. (2021). The Effect of Alendronate on Osteoclastogenesis in Different Combinations of M-CSF and RANKL Growth Factors. Biomolecules.

[10] Trouvin A.P., Goëb V. (2010). Receptor activator of nuclear factor-κB ligand and osteoprotegerin: Maintaining the balance to prevent bone loss. Clin. Interv. Aging.

[11] Boyce B.F., Xiu Y., Li J., Xing L., Yao Z. (2015). NF-κB-Mediated Regulation of Osteoclastogenesis. Endocrinol. Metab..

[12] Cappariello A., Maurizi A., Veeriah V., Teti A. (2014). The Great Beauty of the osteoclast. Arch. Biochem. Biophys..

[13] Yamashita T., Yao Z., Li F., Zhang Q., Badell I.R., Schwarz E.M., Takeshita S., Wagner E.F., Noda M., Matsuo K. (2007). NF-kappaB p50 and p52 regulate receptor activator of NF-kappaB ligand (RANKL) and tumor necrosis factor-induced osteoclast precursor differentiation by activating c-Fos and NFATc1. J. Biol. Chem..

[14] Aguilar-Toalá J.E., Cruz-Monterrosa R.G., Liceaga A.M. (2022). Beyond Human Nutrition of Edible Insects: Health Benefits and Safety Aspects. Insects.

[15] Hirose Y., Ohta E., Kawai Y., Ohta S. (2013). Dorsamin-A’s, glycerolipids carrying a dehydrophenylalanine ester moiety from the seed-eating larvae of the bruchid beetle Bruchidius dorsalis. J. Nat. Prod..

[16] Shi Y.N., Tu Z.C., Wang X.L., Yan Y.M., Fang P., Zuo Z.L., Hou B., Yang T.H., Cheng Y.X. (2014). Bioactive compounds from the insect Aspongopus chinensis. Bioorganic Med. Chem. Lett..

[17] Lee W., Lee J., Kulkarni R., Kim M.A., Hwang J.S., Na M., Bae J.S. (2016). Antithrombotic and antiplatelet activities of small-molecule alkaloids from *Scolopendra subspinipes mutilans*. Sci. Rep..

[18] Van Itterbeeck J., van Huis A. (2012). Environmental manipulation for edible insect procurement: A historical perspective. J. Ethnobiol. Ethnomedicine.

[19] Yeo H., Youn K., Kim M., Yun E.Y., Hwang J.S., Jeong W.S., Jun M. (2013). Fatty Acid Composition and Volatile Constituents of *Protaetia brevitarsis larvae*. Prev. Nutr. Food Sci..

[20] Park Y.M., Noh E.M., Lee H.Y., Shin D.Y., Lee Y.H., Kang Y.G., Na E.J., Kim J.H., Yang H.J., Kim M.J. (2021). Anti-diabetic effects of *Protaetia brevitarsis* in pancreatic islets and a murine diabetic model. Eur. Rev. Med. Pharmacol. Sci..

[21] Yoo Y.C., Shin B.H., Hong J.H., Lee J., Chee H.Y., Song K.S., Lee K.B. (2007). Isolation of fatty acids with anticancer activity from Protaetia brevitarsis larva. Arch. Pharm. Res..

[22] Kang M., Kang C., Lee H., Kim E., Kim J., Kwon O., Lee H., Kang H., Kim C., Jang H. (2012). Effects of fermented aloe vera mixed diet on larval growth of *Protaetia brevitarsis seulensis* (Kolbe) (Coleopteran: Cetoniidae) and protective effects of its extract against CCl4-induced hepatotoxicity in SpragueDawley rats. Entomol. Res..

[23] Nam H.H., Kang S., Seo Y.S., Lee J., Moon B.C., Lee H.J., Lee J.H., Kim B., Lee S., Kim J.S. (2022). Protective effects of an aqueous extract of *Protaetia brevitarsis seulensis larvae* against radiation-induced testicular injury in mice. Food Sci. Nutr..

[24] Choi R.Y., Kim I.W., Ji M., Paik M.J., Ban E.J., Lee J.H., Hwang J.S., Kweon H., Seo M. (2023). *Protaetia brevitarsis seulensis larvae* ethanol extract inhibits RANKL-stimulated osteoclastogenesis and ameliorates bone loss in ovariectomized mice. Biomed. Pharmacother..

[25] Ganguly K., Jeong M.S., Dutta S.D., Patel D.K., Cho S.J., Lim K.T. (2020). Protaetia brevitarsis seulensis Derived Protein Isolate with Enhanced Osteomodulatory and Antioxidative Property. Molecules.

[26] Jang H.Y., Lee H.S., Noh E.M., Kim J.M., You Y.O., Lee G., Koo J.H., Lim H., Ko S., Kim J.S. (2021). Aqueous extract of Chrysanthemum morifolium Ramat. inhibits RANKL-induced osteoclast differentiation by suppressing the c-fos/NFATc1 pathway. Arch. Oral Biol..

[27] Livak K.J., Schmittgen T.D. (2001). Analysis of relative gene expression data using real-time quantitative PCR and the 2(-Delta Delta C(T)) Method. Methods.

[28] Hanley D.A., McClung M.R., Davison K.S., Dian L., Harris S.T., Miller P.D., Lewiecki E.M., Kendler D.L., Writing Group for the Western Osteoporosis Alliance (2017). Western Osteoporosis Alliance Clinical Practice Series: Evaluating the Balance of Benefits and Risks of Long-Term Osteoporosis Therapies. Am. J. Med..

[29] Barasch A., Cunha-Cruz J., Curro F.A., Hujoel P., Sung A.H., Vena D., Voinea-Griffin A.E., Beadnell S., Craig R.G., DeRouen T. (2013). Risk factors for osteonecrosis of the jaws: A case-control study from the CONDOR Dental PBRN. Tex. Dent. J..

[30] Grodstein F., Stampfer M.J., Goldhaber S.Z., Manson J.E., Colditz G.A., Speizer F.E., Willett W.C., Hennekens C.H. (1996). Prospective study of exogenous hormones and risk of pulmonary embolism in women. Lancet.

[31] Narod S.A. (2011). Hormone replacement therapy and the risk of breast cancer. Nat. Rev. Clin. Oncol..

[32] Fontana A., Spolaore B., Polverino de Laureto P. (2013). The biological activities of protein/oleic acid complexes reside in the fatty acid. Biochim. Biophys. Acta.

[33] Calder P.C. (2015). Functional Roles of Fatty Acids and Their Effects on Human Health. JPEN J. Parenter. Enter. Nutr..

[34] Cardoso G.B., Chacon E., Chacon P.G., Bordeaux-Rego P., Duarte A.S., Saad S.T.O., Zavaglia C.A., Cunha M.R. (2017). Fatty acid is a potential agent for bone tissue induction: In vitro and in vivo approach. Exp. Biol. Med..

[35] Fonolla-Joya J., Reyes-García R., García-Martín A., López-Huertas E., Muñoz-Torres M. (2016). Daily Intake of Milk Enriched with n-3 Fatty Acids, Oleic Acid, and Calcium Improves Metabolic and Bone Biomarkers in Postmenopausal Women. J. Am. Coll. Nutr..

[36] Sims N.A., Walsh N.C. (2012). Intercellular cross-talk among bone cells: New factors and pathways. Curr. Osteoporos. Rep..

[37] Arai F., Miyamoto T., Ohneda O., Inada T., Sudo T., Brasel K., Miyata T., Anderson D.M., Suda T. (1999). Commitment and differentiation of osteoclast precursor cells by the sequential expression of c-Fms and receptor activator of nuclear factor kappaB (RANK) receptors. J. Exp. Med..

[38] Suda T., Takahashi N., Udagawa N., Jimi E., Gillespie M.T., Martin T.J. (1999). Modulation of osteoclast differentiation and function by the new members of the tumor necrosis factor receptor and ligand families. Endocr. Rev..

[39] Park J.H., Lee N.K., Lee S.Y. (2017). Current Understanding of RANK Signaling in Osteoclast Differentiation and Maturation. Mol. Cells.

[40] Kim K., Lee S.H., Ha Kim J., Choi Y., Kim N. (2008). NFATc1 induces osteoclast fusion via up-regulation of Atp6v0d2 and the dendritic cell-specific transmembrane protein (DC-STAMP). Mol. Endocrinol..

[41] Lee K., Seo I., Choi M.H., Jeong D. (2018). Roles of Mitogen-Activated Protein Kinases in Osteoclast Biology. Int. J. Mol. Sci..

[42] Lee K., Chung Y.H., Ahn H., Kim H., Rho J., Jeong D. (2016). Selective Regulation of MAPK Signaling Mediates RANKL-dependent Osteoclast Differentiation. Int. J. Biol. Sci..

[43] Kobayashi N., Kadono Y., Naito A., Matsumoto K., Yamamoto T., Tanaka S., Inoue J. (2001). Segregation of TRAF6-mediated signaling pathways clarifies its role in osteoclastogenesis. EMBO J..

[44] Huh J.E., Lee W.I., Kang J.W., Nam D., Choi D.Y., Park D.S., Lee S.H., Lee J.D. (2014). Formononetin attenuates osteoclastogenesis via suppressing the RANKL-induced activation of NF-κB, c-Fos, and nuclear factor of activated T-cells cytoplasmic 1 signaling pathway. J. Nat. Prod..

[45] Abu-Amer Y. (2013). NF-κB signaling and bone resorption. Osteoporos. Int..

[46] Dong M., Zeng J., Yang C., Qiu Y., Wang X. (2022). Asiatic Acid Attenuates Osteoporotic Bone Loss in Ovariectomized Mice Through Inhibiting NF-kappaB/MAPK/ Protein Kinase B Signaling Pathway. Front. Pharmacol..

[47] Shi L., Zhao S., Chen Q., Wu Y., Zhang J., Li N. (2018). Crocin inhibits RANKL-induced osteoclastogenesis by regulating JNK and NF-κB signaling pathways. Mol. Med. Rep..

[48] Deng W., Huang Y., Li H., Chen C., Lin Y., Wang M., Huang H., Liu T., Qin Q., Shao Y. (2022). Dehydromiltirone inhibits osteoclast differentiation in RAW264.7 and bone marrow macrophages by modulating MAPK and NF-κB activity. Front. Pharmacol..

[49] Xu J., Wu H.F., Ang E.S., Yip K., Woloszyn M., Zheng M.H., Tan R.X. (2009). NF-kappaB modulators in osteolytic bone diseases. Cytokine Growth Factor Rev..

[50] Takayanagi H., Kim S., Koga T., Nishina H., Isshiki M., Yoshida H., Saiura A., Isobe M., Yokochi T., Inoue J. (2002). Induction and activation of the transcription factor NFATc1 (NFAT2) integrate RANKL signaling in terminal differentiation of osteoclasts. Dev. Cell.

[51] Asagiri M., Takayanagi H. (2007). The molecular understanding of osteoclast differentiation. Bone.

[52] Tanaka S., Nakamura K., Takahasi N., Suda T. (2005). Role of RANKL in physiological and pathological bone resorption and therapeutics targeting the RANKL-RANK signaling system. Immunol. Rev..

[53] Arai A., Mizoguchi T., Harada S., Kobayashi Y., Nakamichi Y., Yasuda H., Penninger J.M., Yamada K., Udagawa N., Takahashi N. (2012). Fos plays an essential role in the upregulation of RANK expression in osteoclast precursors within the bone microenvironment. J. Cell Sci..

[54] Jeong D.H., Kwak S.C., Lee M.S., Yoon K.H., Kim J.Y., Lee C.H. (2020). Betulinic Acid Inhibits RANKL-Induced Osteoclastogenesis via Attenuating Akt, NF-κB, and PLCγ2-Ca2+ Signaling and Prevents Inflammatory Bone Loss. J. Nat. Prod..

[55] Li S., Miller C.H., Giannopoulou E., Hu X., Ivashkiv L.B., Zhao B. (2014). RBP-J imposes a requirement for ITAM-mediated costimulation of osteoclastogenesis. J. Clin. Investig..

[56] Hwang S.Y., Putney J.W. (2011). Calcium signaling in osteoclasts. Biochim. Biophys. Acta.

[57] Mao D., Epple H., Uthgenannt B., Novack D.V., Faccio R. (2006). PLCgamma2 regulates osteoclastogenesis via its interaction with ITAM proteins and GAB2. J. Clin. Investig..

