# Supplementary material for: Protaetia brevitarsis Extract Attenuates RANKL-Induced Osteoclastogenesis by Inhibiting the JNK/NF-κB/PLCγ2 Signaling Pathway
Source: Nutrients. 2023 Jul 19;15(14):3193. doi: 10.3390/nu15143193 (PMC10383183; doi:10.3390/nu15143193)
Supplement: Supplementary file 1 [file nutrients-15-03193-s001.zip › nutrients-2490568-supplementary.pdf]

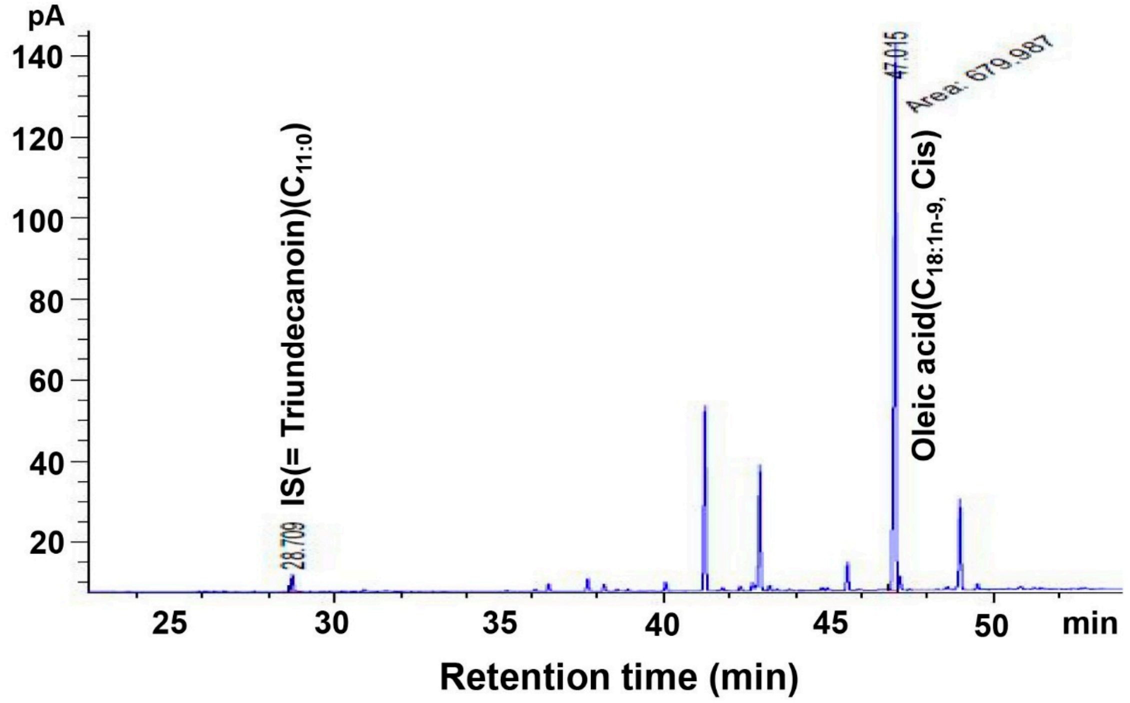

Supplementary Figure S1. Gas chromatography profile of *Protaetia brevitarsis*. Oleic acid was detected.

GC Analysis of *P. Brevitarsis*

The larvae powder was subjected to GC profiling to analyze the constituents present in *P. brevitarsis*. The *P. brevitarsis* used in this study contained a large amount of oleic acid (suppl. Figure S1).
